# Study of Bladder Cancer Detection in Standard White Light Versus AI-Supported Endoscopy-02 (RAISE-02)—A Randomized Controlled Non-Inferiority Trial

**DOI:** 10.3390/cancers18111739

**Published:** 2026-05-26

**Authors:** Peter Blak Hjort, Katharina Skovhus, Jørgen Bjerggaard Jensen, Andreas Ernst

**Affiliations:** 1 Department of Clinical Medicine, Aarhus University, Palle Juul-Jensens Blvd. 82, 8200 Aarhus N, Denmark; katans@rm.dk (K.S.); jbj@jbsquared.dk (J.B.J.); andrerns@rm.dk (A.E.); 2 Department of Urology, Aarhus University Hospital, Palle Juul-Jensens Blvd. 35, 8200 Aarhus N, Denmark; 3 Greenland Centre for Health Research, Institute for Health and Nature, Ilisimatusarfik/University of Greenland, Nuuk 3905, Greenland

**Keywords:** artificial intelligence, bladder cancer, detection, diagnosis, sensitivity

## Abstract

Background: White light cystoscopy (WLC) is the gold standard for bladder cancer (BC) detection and surveillance, but has limited sensitivity, particularly for small and flat lesions. We evaluated the diagnostic performance and safety of a real-time artificial intelligence (AI)-assisted support tool (CystoAID©) for bladder cancer detection. Methods: This single-center, randomized, controlled non-inferiority trial included 64 patients undergoing transurethral resection of bladder tumor or laser fulguration for suspected primary or recurrent BC. Patients were randomized 1:1 to WLC alone or WLC followed by AI-assisted cystoscopy. The primary outcome was per-lesion sensitivity of CystoAID vs. WLC within the intervention group. The reference standard was histopathology or clinical evaluation. The predefined non-inferiority margin was −5%. Secondary outcomes included sensitivity for lesions ≤ 5 mm, false positives, procedural duration, and safety (30-day adverse events). Results: A total of 142 lesions were identified (84 intervention, 58 control). Sensitivity was 96.2% (95% CI 87.0–99.5) for CystoAID and 88.7% (95% CI 77.0–95.7) for WLC (difference 7.5%; 95% CI −2.7 to 17.8), demonstrating non-inferiority. In a sub-analysis for lesions ≤ 5 mm (*n* = 25), sensitivity was 100% (95% CI 86.3–100) vs. 80% (95% CI 59.3–93.2). AI assistance added a median of 2.1 min without further affecting workflow. Adverse event rates were low and similar between groups. Conclusions: CystoAID demonstrated non-inferior sensitivity to WLC and could be safely integrated into the routine clinical workflow. Larger studies with improved representation of flat lesions are warranted, as well as to assess whether CystoAID can achieve superior sensitivity compared with standard WLC.

## Simple Summary

Bladder cancer is one of the most common cancers worldwide and remains a major clinical challenge, particularly with regard to diagnosis and long-term follow-up. Accurate detection of bladder tumors is essential, as missed lesions may lead to delayed treatment and potentially worse outcomes. Consequently, several technologies aimed at improving diagnostic accuracy are currently being investigated, including artificial intelligence (AI)-based tools. In this study, we evaluated an AI-assisted software designed to support cystoscopic detection of bladder cancer lesions. We found that the software was non-inferior to standard diagnostic practice in detecting bladder lesions, indicating that its diagnostic performance was comparable to current methods. In addition, the software could be successfully integrated into the clinical workflow without compromising patient safety or disrupting routine clinical practice.

## 1. Introduction

Bladder cancer (BC) remains one of the most prevalent malignancies worldwide and continues to pose substantial diagnostic and therapeutic challenges in urological practice [1,2]. White light cystoscopy (WLC) is the gold standard for the detection, diagnosis, and surveillance of BC [3,4,5]. Despite this, WLC is limited by suboptimal sensitivity, particularly for flat lesions such as carcinoma in situ (CIS) and small papillary tumors [6,7]. These diagnostic limitations are reflected in the considerable rates of residual disease following transurethral resection of bladder tumors (TURBTs) and high risks of recurrence and progression in non-muscle invasive bladder cancer (NMIBC) [8,9,10,11,12,13].

Tumor visualization enhancement techniques, including photodynamic diagnosis (PDD) and narrow-band imaging (NBI), have demonstrated improved detection compared with WLC alone and are recommended in current European guidelines when available [3,6,14,15]. In a large meta-analysis, they were found to have a pooled sensitivity of 93% and 96%, respectively [6]. However, their adoption is limited by cost, availability, and workflow complexity [16,17]. WLC remains the predominant diagnostic modality in routine clinical practice [18].

Artificial intelligence (AI) is increasingly being applied across the medical sciences; however, in urology, its use for bladder cancer detection remains at a preliminary stage [19,20,21]. While retrospective studies report high diagnostic performance, these results may overestimate real-world effectiveness [22,23,24]. Prospective clinical trials are essential to determine whether AI-assisted cystoscopy can be safely integrated without compromising diagnostic performance [25].

We have previously reported on the development, training, and external validation of the software CystoAID [26]. This randomized non-inferiority clinical trial aimed to evaluate the clinical applicability and safety of AI-assisted cystoscopy with CystoAID as an adjunct to WLC in diagnosing BC.

## 2. Materials and Methods

### 2.1. Study Design

This was a single-center, randomized, controlled non-inferiority trial conducted between November 2024 and September 2025. Patients were referred from three hospitals in Denmark, comprising two regional hospitals and one university hospital. All investigations took place at Aarhus University Hospital.

### 2.2. Participants and Data

All patients referred for flexible cystoscopy due to primary suspicion of bladder cancer or as follow-up for NMIBC were screened for eligibility in the outpatient clinics. Eligible patients were aged 18 years or older and had suspected primary or recurrent bladder cancer. Exclusion criteria included the absence of a bladder tumor and the inability to provide written informed consent.

### 2.3. Randomization

Randomization took place immediately after inclusion in the outpatient clinic. Patients were referred for either TURBT in a day care setting for patients with primary suspicion of bladder cancer, or flexible cystoscopy with laser fulguration in an outpatient setting for follow-up patients. Patients were randomized in a 1:1 ratio between the control group and the intervention group. To ensure balanced allocation, stratified block randomization was used, with stratification according to surgical modality in block sizes of 4 or 6. The allocation list was generated by an external data manager at Aarhus University Hospital.

The clinician performing either the TURBT or laser fulguration was blinded to the allocation until completion of the initial WLC. This ensured that bladder cancer detection during WLC remained unaffected.

### 2.4. Study Procedure

All patients first underwent a standard WLC for initial BC detection. Upon completion of the WLC, as determined by the operating surgeon, the randomization allocation was revealed.

Patients randomized to the control group proceeded directly to treatment according to their planned procedure (TURBT or laser fulguration).

Patients randomized to the intervention group subsequently underwent an additional cystoscopy assisted by CystoAID (version 1.1.0-rc.1), after which the study procedure was completed. During the AI-assisted cystoscopy, CystoAID provided real-time visual overlays highlighting suspected lesions on the cystoscopy monitor. CystoAID was used as a decision-support tool and did not replace clinician judgment. All surgeons performing procedures were either board-certified urologists or senior residents with at least 3 years of experience in urology, and received standardized training in the use of CystoAID.

During all WLCs, lesions identified as malignant were annotated on a standardized bladder map (Supplementary Table S1). Additionally, lesion characteristics, including size (visual assessment), multifocality, suspected T stage, biopsy status, and procedure duration, were recorded. Lesions detected during CystoAID-assisted cystoscopy were annotated on the same bladder map, allowing direct lesion-by-lesion comparison between modalities.

Histopathological staging served as the reference standard when biopsies were available. If the surgeon deemed biopsies unnecessary, the clinical assessment was used as the reference standard.

To ensure accordance with the Danish standard of care, NBI was available at the treating surgeon’s discretion in both study groups. A fixed, locked version of CystoAID 1.1.0-rc.1 was used throughout the study, with no model updates or retraining during the trial. Additional information on CystoAID development, training, hardware specifications, and external validation results has been previously published [26]. The system consists of a hardware unit connected directly to both the cystoscopy monitor and the cystoscope, along with a foot pedal for on/off control. The model was trained on both still images and video sequences with benign, non-malignant pathological, and malignant lesions with histopathological confirmation.

### 2.5. Follow-Up

All adverse events occurring during the procedure or within 30 days after discharge were recorded and graded according to the modified Urological Clavien–Dindo classification (Supplementary Table S2) [27].

### 2.6. Outcomes

The primary outcome was the sensitivity of CystoAID, evaluated in a non-inferiority framework using a predefined non-inferiority margin (see below). Sensitivity was evaluated as the number of malignant lesions identified by WLC and CystoAID, respectively, divided by the total number of malignant lesions identified. Histological reports served as the gold standard, and, in cases with no histology, the treating surgeon served as the gold standard. The primary outcome analysis was restricted to the intervention group, with patients serving as their own comparator.

Secondary outcomes included sensitivity for detection of lesions < 5 mm, number of false positives at lesion level, procedural duration with and without CystoAID assistance, and safety.

### 2.7. Statistical Methods

Baseline characteristics were summarized using medians and 25th–75th percentiles for continuous variables and counts with percentages for categorical variables.

McNemar’s test for paired proportions was used to evaluate sensitivity within the intervention group. Secondary outcomes, except safety, were also analyzed only within the intervention group using paired statistical tests, including McNemar’s test or the Wilcoxon signed-rank test, as appropriate. False positives at the lesion level were assessed as counts in each group.

Safety outcomes were analyzed by comparing the incidence of adverse events between the control and intervention groups. Between-group differences were assessed using Fisher’s exact test.

The non-inferiority margin was set at 5% based on a large meta-analysis reporting a WLC sensitivity of 71%, with a confidence interval spanning ±5% [6]. Preclinical testing of CystoAID demonstrated an estimated sensitivity of 90%. Assuming 80% power, a total sample size of 64 patients (32 per group) was required.

All analyses were conducted on an intention-to-treat basis using R version 4.5.1, R Core Team (2025). *R: A Language and Environment for Statistical Computing*. R Foundation for Statistical Computing, Vienna, Austria. https://www.r-project.org/. All study data were stored on a secure local REDCap database hosted by Aarhus University [28].

### 2.8. Ethics

The study was approved by the National Scientific Ethics Committee (approval no. 2401420, date: 6 September 2024) and the Danish Medicines Agency (approval no. 2024052646, date: 24 September 2024) and registered at clinicaltrials.gov (NCT06780358, date: 13 January 2025). The trial was conducted in accordance with the Declaration of Helsinki and monitored by the local Good Clinical Practice Unit. Written informed consent was obtained from all participants before inclusion. CystoAID is a proprietary medical device and was evaluated under a research agreement with the manufacturer, Cystotech ApS.

## 3. Results

### 3.1. Enrollment

**Figure 1:**
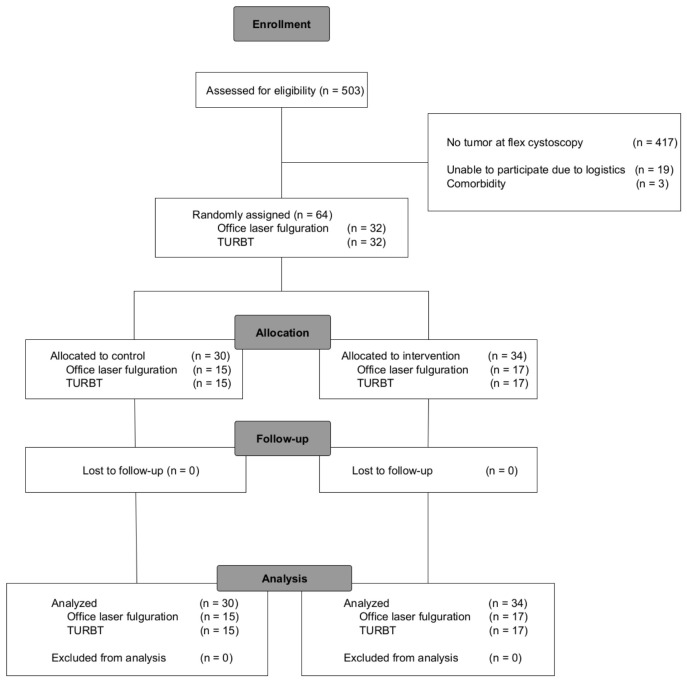
Consolidated Standards of Reporting Trials (CONSORT) diagram.

In total, 503 patients were screened for eligibility. After exclusion of patients without visible tumors (417), patients unable to participate (22), 64 patients were enrolled. An equal number of patients were included in each procedural modality (32 for TURBT, 32 for laser fulguration) (Figure 1).

### 3.2. Baseline Characteristics

Baseline characteristics are shown in Table 1. Overall, sex and age differed between the randomization groups, with higher age and fewer females in the control group. T stage distributions were largely comparable, although slightly more advanced stages were observed in the intervention group. Tumor size distributions were largely comparable.

Among 64 patients with 142 lesions, 71 lesions in 51 patients were assessed histologically. Thus, 71 lesions were evaluated by the clinician. Notably, 13 patients had no histology. In most cases, the clinician deemed histological assessment unnecessary.

### 3.3. Performance of CystoAID

In the intervention group, 84 potential lesions were evaluated, of which 46 were biopsied. Thus, 38 lesions were visually evaluated.

**Figure 2:**
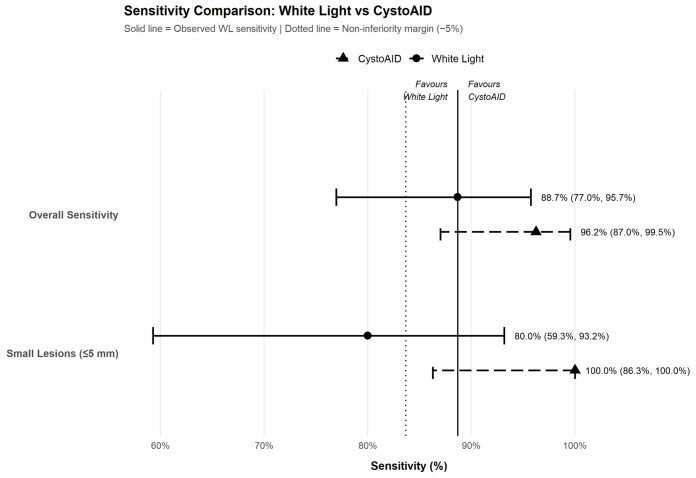
Forest plot of white light sensitivity compared with CystoAID, overall and small lesions.

The detection of tumors using CystoAID was non-inferior to WLC with a sensitivity of 96.2% (95% CI 87.0–99.5) compared with 88.7% (95% CI 77.0–95.7), yielding a difference of 7.5 percentage points (95% CI −2.7–17.8, *p* = 0.29). In a sub-analysis of 25 malignant lesions ≤ 5 mm in size, CystoAID demonstrated 100% sensitivity (95% CI 86.3–100%) compared with WLC at 80% (95% CI 59.3–93.2), equal to a difference of 20% (95% CI 2.5–37.5, *p* = 0.07). Figure 2 shows the sensitivity and corresponding confidence intervals for all lesions, and for lesions ≤ 5 mm.

On a lesion level, CystoAID produced 27 false-positive results, identifying benign areas as malignant; 14 were histologically confirmed as benign, and 13 were clinically evaluated as benign. For WLC, 12 false positives were registered.

### 3.4. Procedural Duration

Table 2 summarizes the duration of (1) the total procedure, (2) WLC, and (3) CystoAID-assisted cystoscopy stratified according to allocation. The median total procedure duration was 21.9 min (Q1, Q3: 14.1, 26.0) in the intervention group vs. 14.6 min (Q1, Q3: 11.4, 19.6) in controls (*p* = 0.044). White light cystoscopy duration was comparable between groups at 3.3 min (Q1, Q3: 2.5, 4.5) and 3.4 min (Q1, Q3: 3.1, 4.6), respectively. In the intervention group, CystoAID required a median of an additional 2.1 min (Q1, Q3: 1.2, 3.4).

### 3.5. Safety

Two adverse events occurred during all procedures. In the intervention group, a software malfunction caused the cystoscopy monitor to temporarily shut off during one procedure. No patient harm occurred, and the manufacturer corrected the software error. One control group patient experienced hematuria.

Twenty-one adverse events were recorded in 18 patients during follow-up: 9 events in 9 control patients and 12 events in 9 intervention patients (Fisher’s exact test: *p* = 0.79). Five adverse events were classified as serious (four in the intervention group, one in the controls); three of these were unrelated to urologic care, and none were related to CystoAID. Table 3 presents an overview of all adverse events.

## 4. Discussion

To our knowledge, this study represents the first randomized controlled trial evaluating real-time clinical application of an AI-support tool for bladder lesion detection. The sensitivity of the CystoAID system was non-inferior to standard WLC. In addition, CystoAID was safe to use and possible to integrate into clinical practice.

Only one other study has evaluated the real-time use of AI-assisted bladder lesion detection. In 2023, Chang et al. published an abstract describing a study including 55 procedures (cystoscopies and TURBTs), reporting a per-frame sensitivity of 52.9% and a per-frame specificity of 98.8% [29]. The apparent discrepancy between their findings and ours is largely attributable to differences in outcome definitions. Whereas we applied per-lesion metrics, Chang et al. reported per-frame performance. However, per-frame analyses tend to inflate specificity, as the majority of cystoscopic frames depict normal urothelium, increasing the number of true negatives.

Most published studies on AI-based cystoscopy remain retrospective and focus on image classification rather than real-time object detection, limiting direct comparison. In 2021, Wu et al. retrospectively evaluated the Cystoscopy Artificial Intelligence Diagnostic System (CAIDS) using 69,204 still images and reported sensitivities ranging from 87 to 98% [30]. While these sensitivity estimates are comparable to ours, they report high specificities (97–99%) as well. However, they include a large proportion of normal urothelium, analogous to the per-frame specificity discussed above. Furthermore, the retrospective design limits their ability to conclude on real-time clinical applicability.

The false-positive rate of CystoAID at the lesion level was relatively high. Although the system is intended for decision support and does not independently trigger biopsies or treatment, the large number of false positives may potentially influence surgeon behavior. This would lead to increased unnecessary biopsies, thereby increasing the risk of patient morbidity and impacting healthcare costs. With bladder cancer being a highly recurrent disease, limiting the number of unnecessary biopsies is of great importance and highlights the need for further software refinement. Especially concerning thresholds for malignant classification and ultimately sensitivity before clinical implementation. This could be accomplished by training the software on larger datasets with complete histopathological confirmation.

We chose not to include specificity as a primary performance metric, since lesion-level specificity is influenced by verification bias. As healthy urothelium is not biopsied, true negatives are not systematically included in the specificity analysis, thus deflating performance. Alternative definitions, such as including unmarked areas adjacent to tumors in the true negative definition, could yield substantially different estimates. Methodological considerations are generally important when interpreting reported performance metrics and should be considered when comparing sensitivity and specificity across AI-assisted cystoscopy studies.

The sensitivity of CystoAID was high (96.2%), exceeding previously reported sensitivities for WLC, which range from 65 to 85% [3,4]. We did not compare performance with enhanced imaging techniques such as NBI and PDD. Since both NBI and PDD have been shown to greatly outperform WLC, these may have been more relevant comparators [3]. However, as WLC remains the most widely available modality in clinical practice, it was selected as the primary comparator in this study to ensure generalizability across varying clinical setups around the world. Furthermore, both NBI and PDD require specialized equipment, photoactive agents, operator expertise, and are not universally available. In contrast, CystoAID relies solely on high-definition video input and requires minimal training, thereby offering broader applicability across clinical settings. Still, to date, the acquisition cost remains unknown.

We performed a sub-analysis on small lesions, which indicated non-inferiority as well, with a difference of 20% (95% CI 2.5–37.5, *p* = 0.07). However, this analysis was based on only 25 lesions, so no definitive performance estimates can be claimed. Furthermore, the evaluation of size was based on the visual assessment of the surgeon.

Median use time was 2.2 min (Q1, Q3: 1.1–3.8), replacing rather than extending standard WLC and maintaining procedural efficiency during laser and TURBT. However, duration may be slightly underestimated, as an initial WLC inspection was performed prior to CystoAID assessment.

The design, a randomized controlled trial, was specifically chosen to evaluate safety. Adverse events were low and comparable between groups, with only one event considered software-related. Overall, we observed no indication that CystoAID compromised patient safety. Randomization ensured that surgeons remained blinded to allocation until after WLC, reducing the risk of performance bias. Assessment of the primary outcome within the intervention group ensured that lesions evaluated by WLC and CystoAID were identical. Inclusion of both newly referred patients and those under surveillance for NMIBC allowed evaluation across flexible and rigid cystoscopy and in the presence or absence of prior resection sites.

Several limitations warrant consideration. The non-inferiority design prevents us from claiming superiority over WLC, even though the results indicate so. The modest sample size led to some baseline imbalance, especially for sex, age, and tumor distribution. Since our primary endpoint was a paired analysis on the intervention group, selection bias should be limited in the primary analysis. However, the baseline imbalances could potentially cause selection bias in our safety assessment. Since there were few adverse events and they were unrelated to the software, we believe the software could be deemed safe regardless of baseline imbalances. Furthermore, the small sample size prevented any conclusions from being drawn based on the sub-analysis on small lesions. The primary outcome, being based on a paired analysis, limits potential selection bias in sensitivity estimates. Nevertheless, the lack of CIS and flat lesions included may have limited external validity. Additionally, procedural time and safety could be affected, but there were no meaningful differences in baseline characteristics when comparing adverse events, and the procedural time difference was mainly driven by laser ablation.

Since the software has not yet been clinically evaluated, we could not obtain permission to perform biopsies on all lesions highlighted by CystoAID, and had to rely on the surgeon’s final evaluation. Furthermore, in cases of multiple tumors, surgeons in Denmark typically perform one representative biopsy and resect or perform laser ablation on the remaining areas. Therefore, histopathological confirmation could not be obtained on all CystoAID markings. This introduces a potential source of verification bias, as unbiopsied CystoAID markings were treated as true positives. If some of these lesions were in fact benign, the true positive rate of CystoAID would be lower than reported, and this should be considered when interpreting our findings. The comparative impact between modalities is, however, likely limited, as it would require the unbiopsied lesions to have been missed entirely during the preceding white light cystoscopy.

CIS and small tumors were under-represented compared with known tumor distribution and generally few, limiting robust evaluation of performance in these clinically important lesion types. This may reflect the study design, in which the inclusion criteria were a visual tumor at the initial white light flexible cystoscopy, known for its risk if missing CIS lesions. This would result in a selected population towards primarily papillary lesions, which could potentially overestimate sensitivity by basing performance estimates on tumors generally detected by WLC. Consequently, to determine CystoAID’s performance on CIS and small lesions, further studies need to be performed with strict lesion size verification and with larger sample sizes.

Together, the small sample size, the lack of histopathological confirmation, and the low number of CIS and flat lesions mean the results and clinical transferability should be cautiously evaluated.

Future studies should include larger, multicenter cohorts; direct comparisons with enhanced visualization techniques; and histological confirmation for all lesions when feasible. Further investigation is also needed to determine whether AI-assisted detection improves re-section completeness or predicts histopathological features. Critically, the long-term impact of AI-supported cystoscopy on recurrence and progression must be evaluated. The utilization of AI in cancer management is transitioning towards multimodal integration, and in the future, CystoAID will probably be combined with several other AI solutions. Specifically, pathology, imaging, and molecular profiling by AI frameworks are being studied, and the integration of these different solutions will greatly impact cancer management in the future [31,32,33]. As regulatory frameworks for AI in medicine continue to evolve, ethical considerations—including consent, accountability, transparency, and data protection—will be essential for widespread implementation.

## 5. Conclusions

This randomized controlled non-inferiority study confirmed that the sensitivity of CystoAID, an AI support tool for bladder cancer detection, was non-inferior to white light cystoscopy. Furthermore, it was safe to use and possible to integrate into real-time workflow, both in an outpatient and in a day surgery setting.

## Figures and Tables

**Table 1:** Baseline characteristics, RAISE-02, Denmark (*n* = 64).

	Randomization	
Characteristic	Control *n* = 30 ^1,2^	Intervention *n* = 34 ^1,2^
**Sex**		
Female	3 (10%)	10 (29%)
Male	27 (90%)	24 (71%)
**Age [years]**	79 (66, 84)	74 (68, 79)
**Age above 70**	21 (70%)	22 (65%)
**Previous BC**	18 (60%)	15 (44%)
**T-stage from the study procedure**		
Benign	4 (14%)	9 (26%)
Ta LG	20 (69%)	16 (47%)
Ta HG	2 (6.9%)	1 (2.9%)
CIS	0 (0%)	1 (2.9%)
T1a	1 (3.4%)	2 (5.9%)
T1b	1 (3.4%)	2 (5.9%)
≥T2	1 (3.4%)	3 (8.8%)
**Concomitant CIS**	0 (0%)	1 (2.9%)
**WHO 2004/2016 grading**		
LG	20 (83%)	16 (73%)
HG	4 (17%)	6 (27%)
**Multifocal tumors**	9 (30%)	10 (29%)
**Tumor size [mm]**	20 (7, 30)	9 (5, 30)
**Tumor** ≥ **3 cm**	2 (6.9%)	3 (8.8%)
**Tumor** ≤ **5 mm**	5 (17%)	19 (56%)
**EAU risk stratification**		
Low risk	16 (64%)	12 (52%)
Intermediate risk	7 (28%)	4 (17%)
High risk	2 (8.0%)	7 (30%)
Very high risk	0 (0%)	0 (0%)

^1^ *n* (%); Median (Q1, Q3). ^2^ BC = Bladder cancer, LG = Low grade, HG = High grade.

**Table 2:** Procedural duration, overall and for each modality.

	Control	Intervention	*p*-Value ^2^
	*n* = 30 ^1^	*n* = 34 ^1^	
**Total procedure duration (minutes)**	14.6 (11.4, 19.6)	22.0 (14.0, 26.2)	0.045
**White light duration (minutes)**	3.5 (3.1, 4.6)	3.4 (2.5, 4.5)	0.4
**CystoAID duration (minutes)**	-	2.2 (1.2, 3.4)	-

^1^ Median (Q1, Q3); ^2^ Wilcoxon rank sum test.

**Table 3:** Adverse events and severe adverse events.

	Intervention		Control	
	Laser ^1^	TURBT ^1^	Laser ^1^	TURBT ^1^
**Adverse events**	3 ^2^	5	2	6
*Clavien Dindo grading*				
*Grade 1*	1 (33)	5 (100)	2 (100)	6 (100)
*Grade 2*	1 (33)	-	-	-
**Serious Adverse Events**	0	4	0	1
*Clavien Dindo grading*				
*Grade 1*	-	1 (25)	-	-
*Grade 2*	-	1 (25)	-	-
*Grade 3*	-	-	-	-
*Grade 4*	-	2 (50)	-	1 (100)

^1^ N (%); ^2^ One incident was categorized a “near event” and no harm came to the patient. Hence, Clavien Dindo grading is available for two patients.

## Data Availability

Data sharing is not possible due to Danish legislation.

## Supplementary Materials

The following supporting information can be downloaded at: https://www.mdpi.com/article/10.3390/cancers18111739/s1, Table S1: Bladder map; Table S2: Modified Clavien-Dindo for Urology.

## Abbreviations

The following abbreviations are used in this manuscript:

WLC	White light cystoscopy
BC	Bladder cancer
CIS	Carcinoma in situ
TURBT	Transurethral resection of bladder tumors
NMIBC	Non-muscle invasive bladder cancer
PDD	Photodynamic diagnosis
NBI	Narrow-band imaging
AI	Artificial Intelligence
