# Supplementary material for: Study of Bladder Cancer Detection in Standard White Light Versus AI-Supported Endoscopy-02 (RAISE-02)—A Randomized Controlled Non-Inferiority Trial
Source: Cancers (Basel). 2026 May 26;18(11):1739. doi: 10.3390/cancers18111739 (PMC13255740; doi:10.3390/cancers18111739)
Supplement: Supplementary file 1 [file cancers-18-01739-s001.zip › cancers-4316422-supplementary.pdf]

SUPPLEMENTARY MATERIAL

Title: Study of bladde**r** cancer detection in standard white light versus **AI**-**s**upported **e**ndoscopy-**02** (RAISE02)

Authors: Peter B. Hjort[a,b], Katharina Skovhus[a,b], Jørgen B. Jensen[a,c, d], Andreas Ernst[a,b]

Affiliations:

[a]Department of Clinical Medicine, Aarhus University, Palle Juul-Jensens Blvd. 35, 8200 Aarhus N, Denmark.

[b]Department of Urology, Aarhus University Hospital, Palle Juul-Jensens Blvd. 35, 8200 Aarhus N, Denmark

[c]Cystotech ApS, Inge Lehmanns Gade 10, 5, 8000 Aarhus C

[d]Greenland Centre for Health Research, Institute for Health and Nature, Ilisimatusarfik/University of Greenland, Nuuk, Greenland.

Corresponding author and contact information:

Peter B. Hjort

Address: Palle Juul-Jensens Blvd. 35, 8200 Aarhus N, Denmark.

E-mail: petehj@rm.dk

Phone: +4527647744

ORCID ID: orcid.org/0000-0002-3931-9727

Contents

S1: Bladder map 2

A: Annotation table 2

B: Location illustration 3

S2: Modified Clavien-Dindo for Urology, adapted from Bansal et al. [1] 4

S3: Boxplot of procedure durations, total and for CystoAID and white light cystoscopy respectively, stratified by surgical modality. ....**Error! Bookmark not defined.**

Table S1: Bladder map

A: Annotation table

Patient Label			**Randomization group**	
Date of procedure (DD-MM-YYYY)			Control: *Mark with* ☐	Intervention: *Mark with* ☐
**Time spent in WL-modality** **Minutes** ☐	**Time spent in CystoAID-modality** **Minutes** ☐		**Remaining time spent on surgery/ Standard of Care** **Minutes** ☐	

**Lesion no.**	**Size mm**	**Location**	**Suspected T-stage**	**WL Modality** *Mark with an X*	**CystoAID Modality** *Mark with an X*	**Biopsy performed (yes/ no)** If yes insert the pathology requestion no.	**Lesion detected/ treated without biopsy** *Yes/ No*
**1**							
**2**							
**3**							
**4**							
**5**							
**6**							

**7**							
**8**							
**9**							

B: Location illustration

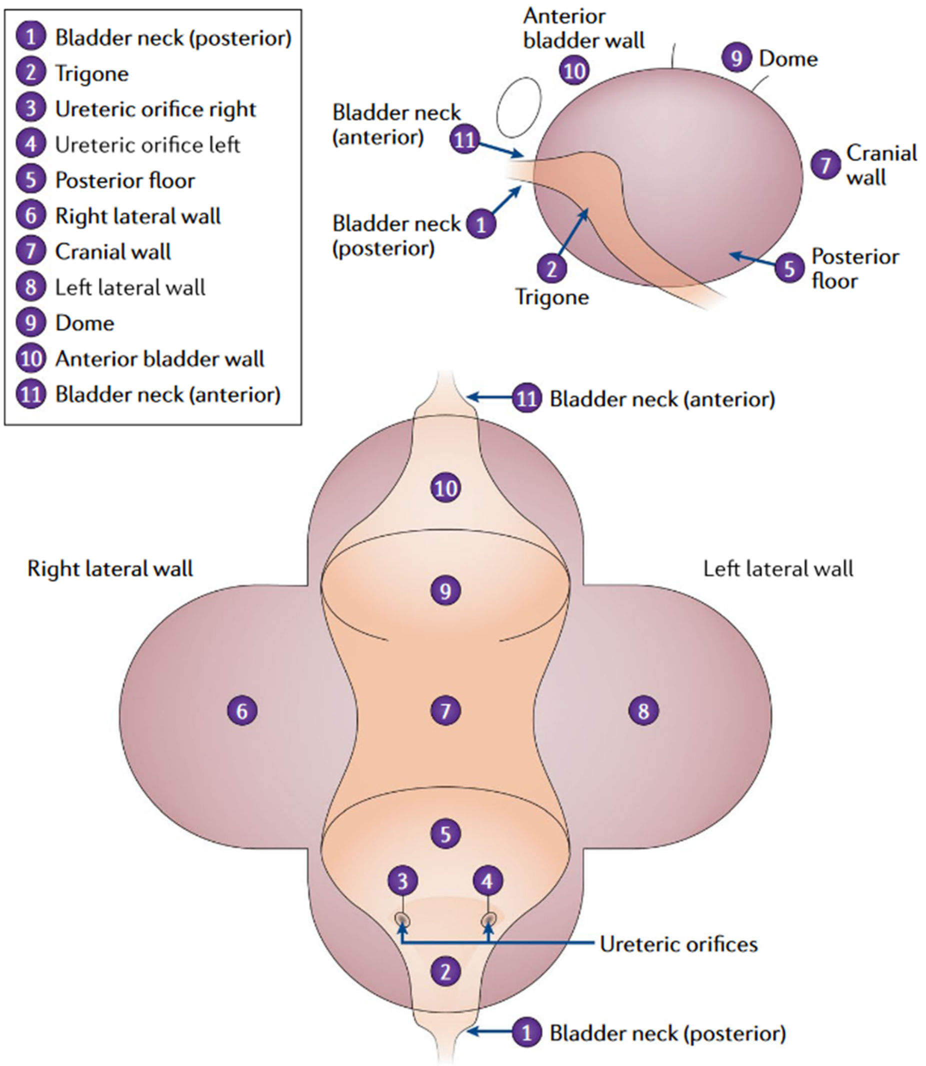

Table S2: Modified Clavien-Dindo for Urology, adapted from Bansal et al. [1]

**Clavien Dindo Scale of Surgical Complications**	
**Grade**	**Definition**
Grade I	Any deviation from the normal postoperative course without the need for therapeutic intervention. This includes minor complications such as wound infections that require only oral antibiotics or urinary tract infections
Grade II	Complications that require pharmacological treatment, such as blood transfusions, intravenous antibiotics, or medications to manage urinary retention.
Grade III	Complications that require surgical, endoscopic, or radiological interventions, but not in a critical care setting. Examples include surgical site infections requiring debridement, bile leaks, or postoperative hemorrhage requiring a return to the operating room.<br>- Grade IIIa: Complications requiring intervention but not requiring general anesthesia. This can include wound debridement, drain placement or endoscopic procedures<br>- Grade IIIb: Complications requiring intervention under general anesthesia. This can include reoperation, percutaneous drainage, or endoscopic procedures.
Grade IV	Complications that require critical care management, such as admission to the intensive care unit (ICU) due to complications like acute renal failure or respiratory failure.<br>- Grade IVa: Life-threatening complications that require ICU management, such as cardiac arrest, acute renal failure, or respiratory failure.<br>- Grade IVb: Complications leading to organ dysfunction requiring surgical intervention. This can include bowel perforation, liver necrosis, or acute lung injury.
Grade V	A severe complication that results in the patient's death.
